# Limit cycle dynamics can guide the evolution of gene regulatory networks towards point attractors

**DOI:** 10.1038/s41598-019-53251-w

**Published:** 2019-11-14

**Authors:** Stuart P. Wilson, Sebastian S. James, Daniel J. Whiteley, Leah A. Krubitzer

**Affiliations:** 1 Department of Psychology, The University of Sheffield, Sheffield, United Kingdom; 2 Center for Neuroscience, University of California, Davis, United States; 3 Department of Psychology, University of California, Davis, United States

**Keywords:** Evolutionary theory, Gene regulatory networks

## Abstract

Developmental dynamics in Boolean models of gene networks self-organize, either into point attractors (stable repeating patterns of gene expression) or limit cycles (stable repeating sequences of patterns), depending on the network interactions specified by a genome of evolvable bits. Genome specifications for dynamics that can map specific gene expression patterns in early development onto specific point attractor patterns in later development are essentially impossible to discover by chance mutation alone, even for small networks. We show that selection for approximate mappings, dynamically maintained in the states comprising limit cycles, can accelerate evolution by at least an order of magnitude. These results suggest that self-organizing dynamics that occur within lifetimes can, in principle, guide natural selection across lifetimes.

## Introduction

Self-organization and natural selection are fundamental forces that shape all biological systems. Self-organization describes a dynamic in a system whereby local interactions between components collectively yield a global order that is unobservable, in its entirety, to the individual components. Therefore, self-organization describes dynamics that occur across all spatial and temporal scales throughout the lifetimes of all organisms. Natural selection is instead a description of dynamics that occur primarily between lifetimes, via the communication of genetic information from organisms to their offspring. It is clear how natural selection can operate on the self-organizing processes by which organisms develop and compete, because information passed on by genetic inheritance specifies the interactions within and between those self-organizing processes. But the extent to which self-organization can operate on natural selection is not yet understood. Can selection, modelled as a global optimization of the genotype by fitness maximization *between lifetimes*, exploit the emergence of structure in the local mapping from genotype to phenotype that occurs *within lifetimes*? Here we show that fitness landscapes can be modified by the intrinsic properties of dynamical network self-organization, via a simple, biologically plausible mechanism that is compatible with conventional descriptions of evolution by natural selection.

**Figure 1:**
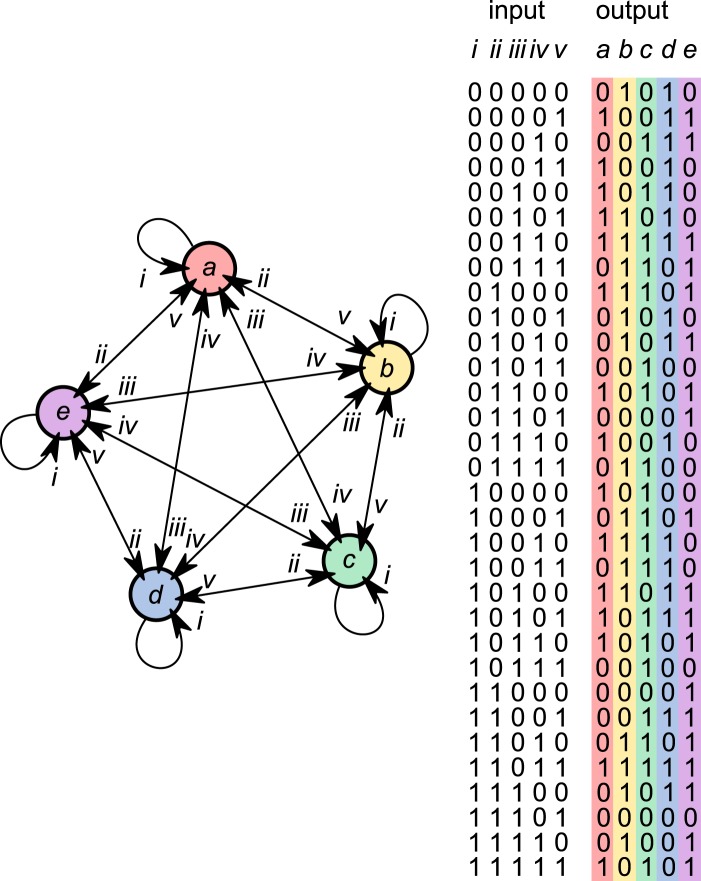
Gene interaction network. A network of $n=5$ interacting genes, shown labelled a–e, each with inputs labelled i–v. The truth table determines the expression level of each gene in response to each of the ${2}^{n}=32$ possible patterns of gene expression. The coloured elements thus constitute a ‘genome’ of $N=n{2}^{n}=160$ bits, which in this case specifies a maximally fit network ($f=1$).

Consider a network of *n* interacting genes and assume for simplicity that their expression levels may be either high or low only. The network interactions can be specified by assigning to each gene a truth table that determines its next expression level in response to each of the 2^*n*^ possible patterns of expression. The developmental dynamics of the network can thus be completely specified by a string comprising $N=n{2}^{n}$ binary digits. Whilst acknowledging the obvious limitations of the analogy, we can, for convenience, refer to this binary string as a ‘genome’. The genome has 2^*N*^ possible configurations. See Fig. 1.

The dynamics in these networks self-organize to reveal attractors^1–3^. From a given initial state (a state is a pattern of *n* binary expression levels), the network activity will eventually settle, either into an endless repetition of a single state, known as a point attractor, or into a limit cycle, where a specific sequence of states repeats endlessly (see^4,5^). In the broadest terms, different initial states represent different contexts in which the network dynamics may develop, as determined by factors extrinsic to the network, such as the transient influence of another gene or gene network, differences between cell or tissue types, or different temperature or chemical conditions (food, oxygen, hormones etc.). Thus we might consider a mapping from a given initial state to a point attractor to constitute a robust response of the network in that context. Assuming that the initial states are determined by such extrinsic factors, the problem for natural selection is to configure an *N*-dimensional genome such that the resulting network interactions will map a given set of initial states to a given set of point attractor states.

**Figure 2:**
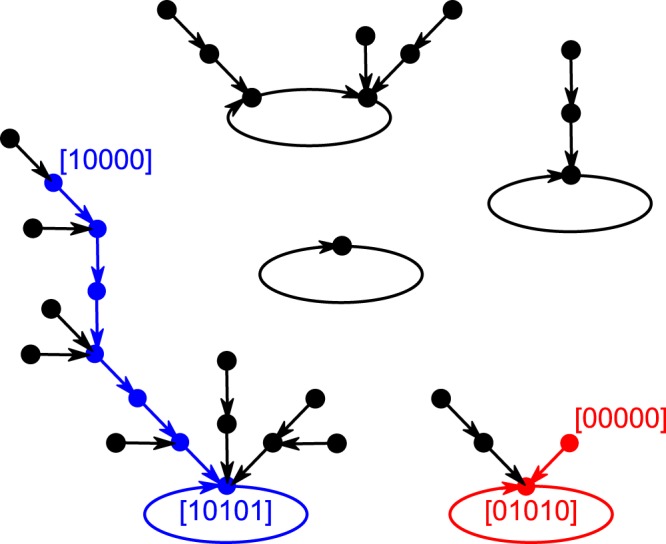
Attractor landscape. The developmental dynamics of the network that is specified by the genome in Fig. 1 reveals five attractors (four point attractors and one with a limit cycle of length two). Every possible gene expression pattern is represented by one dot, and the transitions between states are represented by arrows. Initial states [10000] and [00000] map to target states [10101] and [01010] as point attractors. The blue path corresponds to the development of the network in the anterior context and the red path corresponds to the development of the network in the posterior context.

An instructive example was considered by Giacomantonio & Goodhill^6^, concerning the interactions between genes Fgf8^7,8^, Emx2^9–11^, Pax6^11,12^, Coup-tf1^13,14^, and Sp8^14,15^, which specify position information in the embryonic neocortex and ultimately guide the growth of thalamocortical axons by chemoattraction (e.g.^16,17^; see^18,19^ for reviews). At embryonic day 9.5 (E9.5), before the other transcription factors are known to be expressed, the telencephalic morphogen Fgf8^20^ is secreted only at the anterior neural ridge of the developing forebrain^8^ (see^18^ for a review). Together with other signalling molecules and patterning centers, the secretion of Fgf8 at E9.5 induces the graded expression of Emx2, Pax6, Coup-tf1, and Sp8 in the progenitor cells in the ventricular zone. Interactions between these genes yield posterior to anterior gradients in Emx2 and Coup-tf1 expression and anterior to posterior gradients in Pax6 and Sp8 expression. Hence, in two contexts, defined by the differential expression of Fgf8, $n=5$ genes map initial state [00000] to the target point attractor [01010] in the posterior domain, and map initial state [10000] to the target point attractor [10101] in the anterior domain (binary expression levels ordered as the names of the genes are listed above). See Fig. 2.

By chance mutation alone, the problem of finding a genome configuration to facilitate such developmental dynamics is very difficult. Even for $n=3$ genes, the genome is of length $N=3\times {2}^{3}=24$, and there are ${2}^{N}=16777216$ possible genome configurations. An exhaustive search reveals that 11384 of these possible configurations (0.068%) map differential expression of one gene to differential expression of *n* genes, e.g., mapping initial states [000] and [100] to point attractors [010] and [101]. Current computing power does not allow for an equivalent figure to be determined by an exhaustive search of the genome space for more than three genes. How then has natural selection been able to discover gene-interaction networks that yield stable developmental dynamics, i.e., the emergence of point attractors?

## Results

### Modelling the interaction between self-organization and selection

Discovering the genetic conditions from which specific point attractors can emerge becomes tractable, even for larger networks, if we assume that self-organization, i.e., the emergence of attractor dynamics during development, is able to interact with natural selection in the following way. Starting with a random genome, a network of $n=5$ genes ($N=160$) might from some initial state, e.g., [00000], settle into a particular limit cycle, e.g., [11000] then [00011] then [01011], before repeating [11000] and continuing indefinitely. As the network continues to cycle through these three states, the five genes will be expressed for the following proportions of time: 1/3, 2/3, 0/3, 2/3, 2/3. These values correspond to the relative production rates of five proteins. If the target point attractor state is e.g., [01010], then the protein production levels are ‘correct’ in the following proportions: 2/3, 2/3, 3/3, 2/3, 1/3. The mathematical product of these values thus represents the extent to which downstream processes will be orchestrated by the correct distribution of proteins, and as such it can be used as a measure of the fitness of the genome,

1 $$f=\mathop{\prod }\limits_{x}^{X}\,\mathop{\prod }\limits_{g}^{n}\,(1-\frac{1}{T}\,\mathop{\sum }\limits_{t}^{T}\,|{s^{\prime} }_{g,x}-{s}_{g,x,t}|),$$

where *x* is an index over *X* contexts (each defined by an initial network state), *t* is an index over *T* states comprising the attractor for context *x*, and *s*′ is the target level of gene expression.

In simulation, states *s* can be identified simply by iterating the network dynamics 2^*n*^ times (to guarantee that an attractor is reached) and then iterating a further *T* times until a repetition is detected. Natural selection can then be represented in its simplest terms by flipping each of the *N* genome bits with probability $p\in [0,0.5)$, accepting the modified genome if $\Delta f\ge 0$, and repeating the process for each simulated generation.

It is important to emphasise that according to Eq. (1), the fitness is derived from the developmental dynamics of a network by comparing the target state with the *time average* expression level of each gene, given that the network will cycle through the states in its attractor indefinitely. Hence the model represents an assumption that dynamics in gene networks can propagate quickly with respect to the timescale over which the corresponding protein levels accumulate and interact with downstream processes (see^4,5^). Note that according to Eq. (1), $f=1$ only if all initial states map to the target states as point attractors. Note also that $f=0$ if any gene (*g*) is expressed incorrectly in any attractor state (*s*) that is visited in any context (*x*). Furthermore, note that none of the states in the limit cycle are required to correspond exactly to the target state for the network to be considered to have positive fitness, as is the case for the example considered above. Finally, note that setting $p=0.5$ defines a control condition in which the evolutionary process becomes equivalent to a random sampling of points in the genome space.

### Limit cycle dynamics can guide evolution

**Figure 3:**
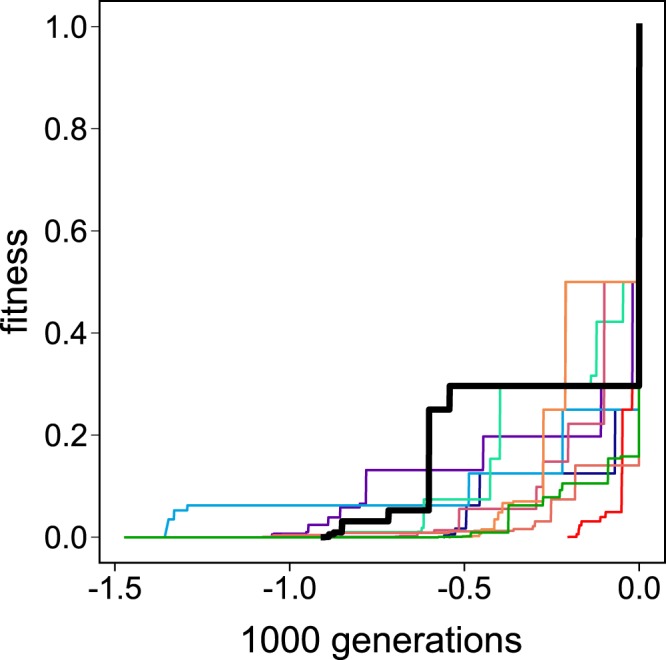
Punctuated equilibria. Evolution of ten genomes by attractor scaffolding (mutation rate $p=0.1$), with the generations at which $f=1$ aligned to zero. Evolution yields long periods of stasis punctuated by sharp fitness increments. The bold trace shows the evolution that gave rise to the network detailed in Figs 1 and 2.

We consider first the example from neocortical development ($n=5$, $X=2$), where initial state [00000] should map to state [01010] as a point attractor, and initial state [10000] should map to state [10101] as a point attractor. Evolving for a total of 10^8^ simulated generations at each mutation rate, from $p=0.05$ to $p=0.45$ at increments of 0.05, revealed dynamics similar to ‘punctuated equilibria’^21,22^, whereby long periods of stasis ($\Delta f=0$) were punctuated by increments in fitness (see Fig. 3). The distribution of the number of generations in each period of stasis is shown in Supplementary Material S1.

**Figure 4:**
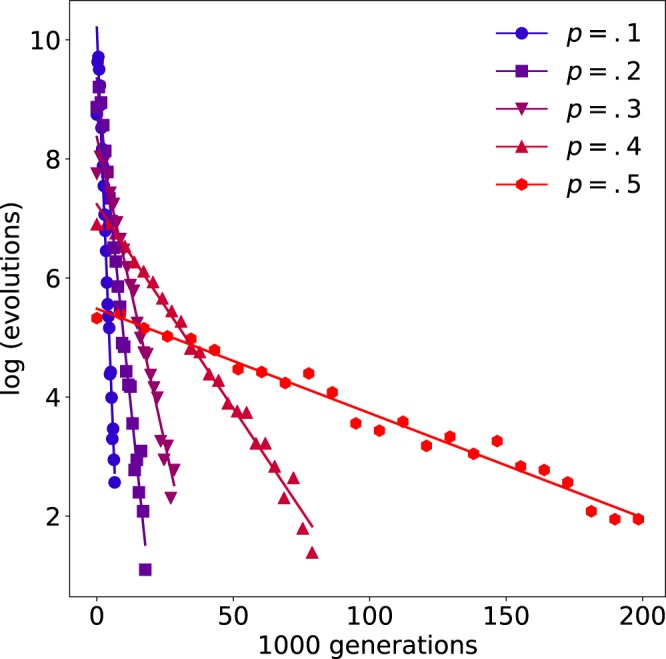
Limit cycle dynamics guide evolution. Distribution of generations required to discover $f=1$ networks at a range of mutation rates *p*. Evolution at lower mutation rates corresponds to searching the fitness landscape more locally, and is shown here to increasingly accelerate the discovery of maximally fit $f=1$ networks. Note that $p=0.5$ corresponds to a random search.

At each mutation rate, the distribution of the number of generations required to discover a maximally fit genome ($f=1$) was long-tailed, conforming increasingly to a log-normal distribution for smaller values of *p*, i.e., for an increasingly local search of the genome space (see Fig. 4). At $p=0.05$, the number of $f=1$ genomes discovered was 70 times greater than by random sampling ($p=0.5$), with discovery taking 853 generations on average. Reducing the mutation rate further (i.e., flipping an average of 6 or less bits per generation) reduced the evolutionary speed-up, confirming that the fitness landscape is not smooth near the fitness peaks. Overall, the average number of generations required to discover $f=1$ networks, *μ*, was well approximated by $\mu (p)={e}^{8.90p+6.22}$ for $p > 0.05$ (for values of *p* less than 0.05, $\mu \to \infty$ as $p\to 1/N$ and $\mu (0)=\infty$).

These results show that self-organization can accelerate selection under the assumption that dynamically stable protein production levels that are more similar to ideal levels yield better fitness. Under this assumption, approximate network solutions that emerge within a lifetime as limit cycle attractors can provide a scaffold of graded fitness around otherwise isolated peaks in the fitness landscape, for natural selection to climb. We thus refer to this mechanism as *attractor scaffolding*. Self-organization can only assist selection via attractor scaffolding if the embedding of attractor landscapes in the *N*-dimensional genome space is locally structured, as is evidenced here by further accelerations in the discovery of $f=1$ genomes at lower mutation rates, i.e., as the search through genome space is more local.

### Fit networks and random networks have equal complexity

Many known biological networks comprise Boolean functions that belong to particular classes of low complexity, such as threshold functions and canalizing functions, i.e., genes for which either expression level is guaranteed by a specific expression level (1 or 0) in at least one other gene^1,2,23,24^. To investigate whether networks generated by attractor scaffolding belong to such classes, we compared the complexity of functions generated in ten thousand $f=1$ networks to the complexity of functions generated in ten thousand randomly configured networks. Following Gherardi & Rotondo^25^, we used the Quine-McCluskey algorithm to derive, for each gene, an equivalent Boolean logic expression (in disjunctive normal form) with the fewest terms, and measured the complexity of each as the number of terms normalised by 2^*n*^. A t-test revealed no significant difference between the mean complexity of Boolean functions in $f=1$ networks (mean = 0.23 ± 0.017) and in randomly generated networks (mean = 0.23 ± 0.017). As we might therefore expect, very few fit networks (0.11%), like random networks (0.11%), contained canalizing functions, which are typically observed in $n=5$ networks for complexities of around 0.1^25^. There was also no difference between fit and random networks in terms of the bias, i.e., the proportion of truth table values that are 1 (mean = 0.50 ± 0.04 versus 0.50 ± 0.04), or the number of attractors (mean = 2.46 ± 1.12 versus mean = 2.43 ± 1.12). Hence we conclude that networks generated by attractor scaffolding are equal in complexity to randomly configured networks.

### Attractor scaffolding is robust to the choice of contexts and integration method

**Figure 5:**
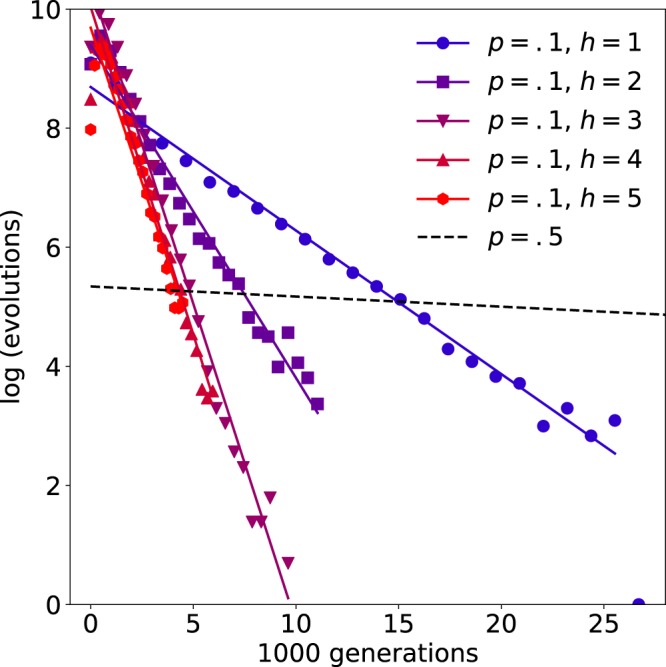
Attractor scaffolding varies with distance between targets. The number of generations required to discover maximally fit networks decreases as the Hamming distance (*h*) between $X=2$ target states increases. Solid lines show fits for distributions obtained using a mutation rate of $p=0.1$. Distributions for each *h* were identical for the $p=0.5$ control (dashed line). The minimum evolutionary speed-up was a factor of 16 (comparing the mean evolutions at $p=0.1$ and $p=0.5$ for $h=1$).

We next investigated whether attractor scaffolding is sensitive to the choice of the initial and target states that define each context. In the example from neocortical development, the initial states differed by one bit (the expression level of the first gene, representing Fgf8) and the target states differed by *n* bits (i.e., defining *n* binary gradients). So we first repeated the original simulations using an anterior initial state that differed from the posterior initial state by a Hamming distance that ranged from 1 (e.g., state [10000] as used originally) to *n* (e.g., [11111]). The choice of the initial state had no effect on the evolutionary dynamics. Next we repeated the original simulations using an anterior target state that differed from the posterior target state by a Hamming distance that ranged from 1 (e.g., state [11010]) to *n* (e.g., [10101] as used originally). Figure 5 shows how attractor scaffolding depends on the distance between the two target states. The number of generations required to discover $f=1$ networks was found to decrease as the Hamming distance between the two target states was increased, with networks mapping from initial states that differed by one bit to target states that differed by one bit discovered in 3574 generations on average, corresponding to an evolutionary speed-up of 16. Note that the choice of target state had no effect on the distribution of discovery times in the $p=0.5$ control condition.

**Figure 6:**
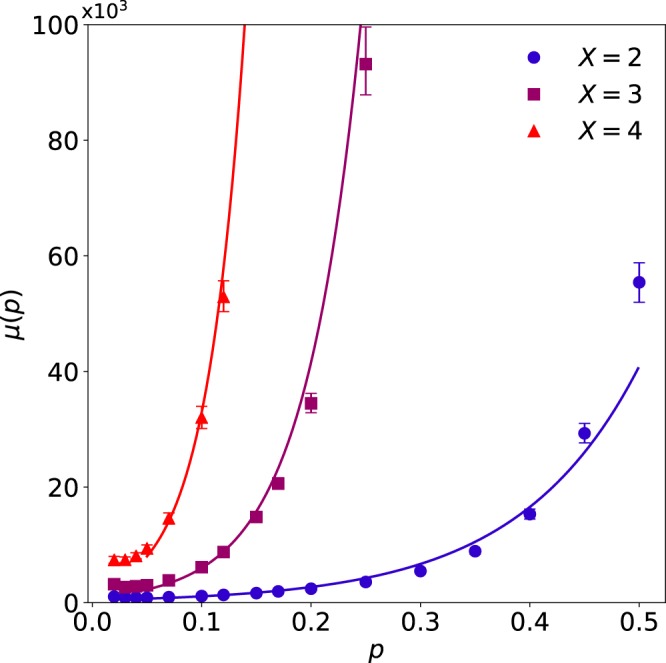
Attractor scaffolding varies with the number of contexts. As the number of contexts *X* increases, more generations are required to discover fit genomes. For each value of *X*, the mean discovery period *μ* increased approximately exponentially with the mutation rate *p* (fits shown by solid lines). Error bars show 95% confidence intervals, determined by a bootstrap analysis of the mean, *μ*, with 1024 resamples.

**Figure 7:**
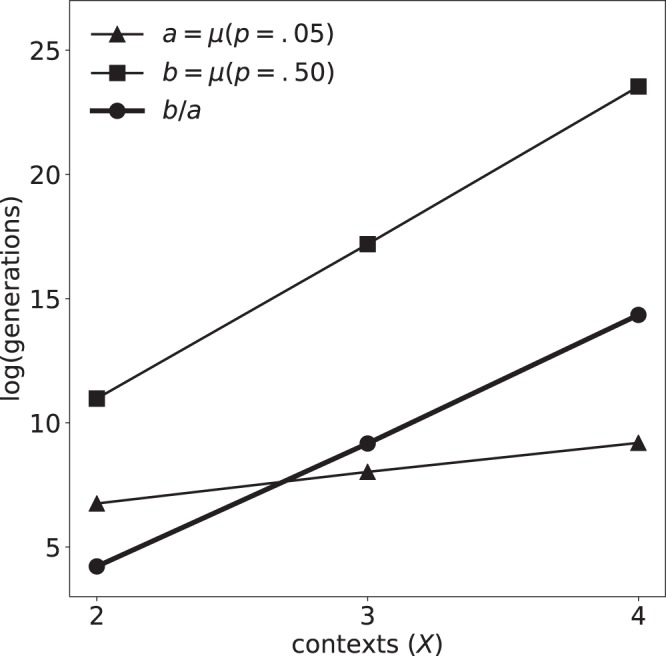
Evolutionary speed-up increases with the number of targets. The maximum average discovery period *b* (for the $p=0.5$ control condition), divided by the minimum average discovery period *a* (for $p=0.05$) yields an estimate of the evolutionary speed-up by attractor scaffolding (circles connected by a thick line), and is here shown to increase exponentially with the number of contexts *X*.

The evolutionary speed-up was also affected by the number of contexts, *X*. For an arbitrary choice of initial and target states (all unique), we ran simulations for $X=2$, $X=3$, and $X=4$ contexts. The simulation was evolved at a range of mutation rates ($p < 0.5$) until 1000 networks were discovered for each combination of *X* and *p*. As the number of contexts was increased, more generations were required to discover fit genomes (see Fig. 6). To obtain sufficient data for analysis, the model was run for the $p=0.5$ control condition through 10^8^ ($X=2$), 5 × 10^9^ ($X=3$), and 2 × 10^11^ ($X=4$) generations. Although the shortest $f=1$ discovery period increased with *X*, the discovery period in the $p=0.5$ control case increased at a far greater rate. Defined as the mean discovery period at $p=0.5$ (where periods were always maximal) divided by the mean discovery period at $p=0.05$ (where periods were always minimal), the evolutionary speed-up by attractor scaffolding *increased* with the number of contexts. Remarkably, the speed-up increased at an approximately exponential rate with the number of contexts (Fig. 7). Thus, as the set of target attractors increased in size, the effectiveness of attractor scaffolding was maintained, despite an exponential increase in the difficulty of the search.

**Figure 8:**
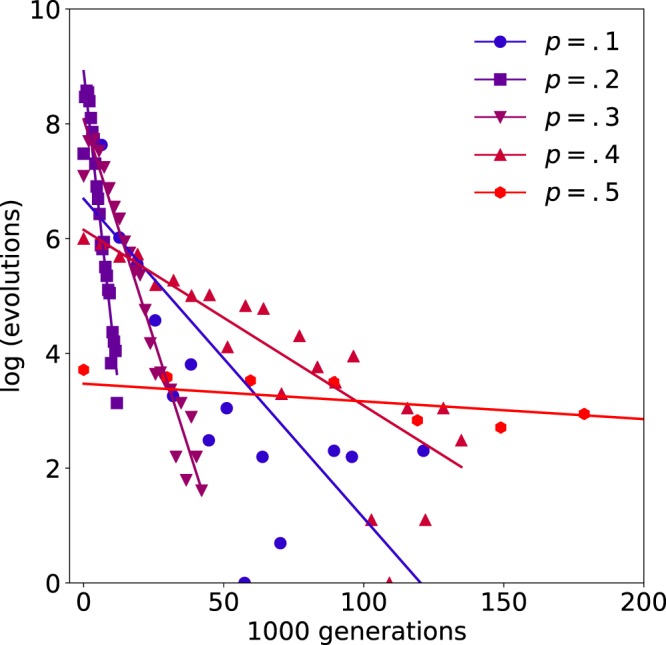
Asynchronous updating. The simulation used to create Fig. 4 was modified to update the state of the network asynchronously. The trend for faster discovery of fit networks at lower mutation rates was comparable, though note that the maximum evolutionary speed-up was obtained at $p=0.15$. Attractor scaffolding is therefore robust to the choice of integration method.

As a final test of robustness, we adapted the original simulation (neocortical example with $X=2$) to instead update the state of the network asynchronously. Instead of changing the states of all *n* genes at the current timestep based on the states of all *n* genes at the previous timestep, asynchronous updating involves iteratively changing the state of one randomly selected gene at a time. To compute the fitness, these dynamics were iterated $T=2N$ times from the initial state in each context and Eq. (1) was calculated with respect to all *XT* visited states; an evolutionary run was terminated when $f > 0.95$. Overall, the average number of generations, *μ*, required to discover fit networks was well approximated by $\mu (p)={e}^{13.60p+5.33}$ for $p > 0.15$ (for values of *p* less than 0.15, $\mu \to \infty$ as $p\to 1/N$ and $\mu (0)=\infty$) (Fig. 8). The effect of attractor scaffolding is therefore robust when the implicit assumptions of a synchronous clock and deterministic network interactions are relaxed.

## Discussion

The effect of self-organization by attractor scaffolding resembles that presented by Hinton & Nowlan^26^. In their seminal model, some genome bits are adaptable within the lifetime of each member of a population, and their genomes are recombined with a probability that decreases with the number of flips of these adaptable bits before a target genome is discovered. The state of adaptable bits is not inherited, but inheritance of the ability to flip state nevertheless increases the discovery rate. Faster discovery of target states within lifetimes therefore directs selection pressure in favour of genetic conditions from which targets can be more rapidly acquired. Attractor scaffolding confers a similar advantage; in both cases an approximation to the target is maintained within the lifetime and communicated only indirectly between lifetimes. An important distinction is that by attractor scaffolding, the benefit of distributing approximate solutions across limit cycle states, rather than across members of a population, is conferred by developmental dynamics intrinsic to individual organisms.

It is interesting that attractor scaffolding was affected by the similarity between the target states. One implication of this result is that networks like that described for cortical arealization, which can be perturbed by the expression of a morphogen into generating orthogonal patterns of stable gene expression, may be more likely to be discovered by natural selection. It is also interesting that the evolutionary speed-up via attractor scaffolding increases exponentially with the number of target attractors. This result suggests that the challenge for an evolutionary search based on iterative evaluation of chance mutations may scale with the complexity of the phenotype (i.e., with properties of the emergent attractor landscape) rather than with the complexity of the genotype (i.e., with the naturally occurring frequency of fit network specifications).

Attractor scaffolding offers a potential mechanism for genetic assimilation; by the gradual evolution of limit cycle dynamics towards point attractor dynamics. Thus, it might support a range of epigenetic phenomena, such as the Baldwin effect(s)^27–30^. Similar principles may help to explain the ‘molecular logic’ of specific gene networks, e.g., networks responsible for the embryonic development of neocortical circuits^6,18,31,32^, and how intrinsic properties of network self-organisation may similarly constrain the evolution and development of functional neuronal networks^33–36^. Practical applications may involve new methods for programming large circuits of logic operations. For example, we found that an $n=7$ circuit, for which the space of configurations comprises $N={2}^{896}$ possibilities, can be configured to robustly map three initial states to three distinct target states in a few million computationally inexpensive steps.

## Methods

A standalone implementation of the model is provided as Supplementary Material S2 and a script for recreating Fig. 4 from the main text is provided as Supplementary Material S3. Copy the text from S2 into a file with a .cpp extension, e.g., evolve.cpp, and copy the text from S3 into a file with a .py extension, e.g., plot.py. From the command line compile using e.g., ‘g++ -O3 evolve.cpp -o evolve’, run the model using ‘./evolve’, then plot using ‘python plot.py’. These programs are part of a full repository of code and additional analysis and visualization tools maintained at https://github.com/ABRG-Models/AttractorScaffolding.

## Supplementary information

Supplementary Material S1, S2, and S3
