# Supplementary material for: Limit cycle dynamics can guide the evolution of gene regulatory networks towards point attractors
Source: Sci Rep. 2019 Nov 14;9:16750. doi: 10.1038/s41598-019-53251-w (PMC6856163; doi:10.1038/s41598-019-53251-w)
Supplement: Supplementary file 1 — Supplementary Material S1, S2, and S3 [file 41598_2019_53251_MOESM1_ESM.pdf]

Limit cycle dynamics can guide the evolution of gene regulatory networks towards point attractors

Stuart P. Wilson[1*], Sebastian S. James[1], Daniel J. Whiteley[1], Leah A. Krubitzer[2,3]

[1]Department of Psychology, The University of Sheffield, Sheffield, United Kingdom.

[2]Center for Neuroscience, University of California, Davis, United States.

[3]Department of Psychology, University of California, Davis, United States.

[*]Corresponding author

Email: S.P.Wilson@Sheffield.ac.uk

Supplementary Figure S1 is a figure showing the distribution of the periods of stasis (where $\Delta f = 0$) for a range of mutation rates p (simulations as in Fig. 3 and Fig. 4 of the main text). The distribution conforms increasingly to a log-normal distribution for lower mutation rates.

Supplementary Material S2 is a standalone implementation of the model in c++. Supplementary Material S3 is a python script for recreating Fig. 4 from the main text.

To recreate Fig. 4 from the main text, copy the text from S2 into a file with a .cpp extension, e.g., evolve.cpp, and copy the text from S3 into a file with a .py extension, e.g., plot.py. From the command line compile using e.g., 'g++ -O3 evolve.cpp -o evolve', run the model using './evolve', then plot using 'python plot.py'. These programs are part of a full repository of code and additional analysis and visualization tools maintained at https://github.com/ABRG-Models/AttractorScaffolding.

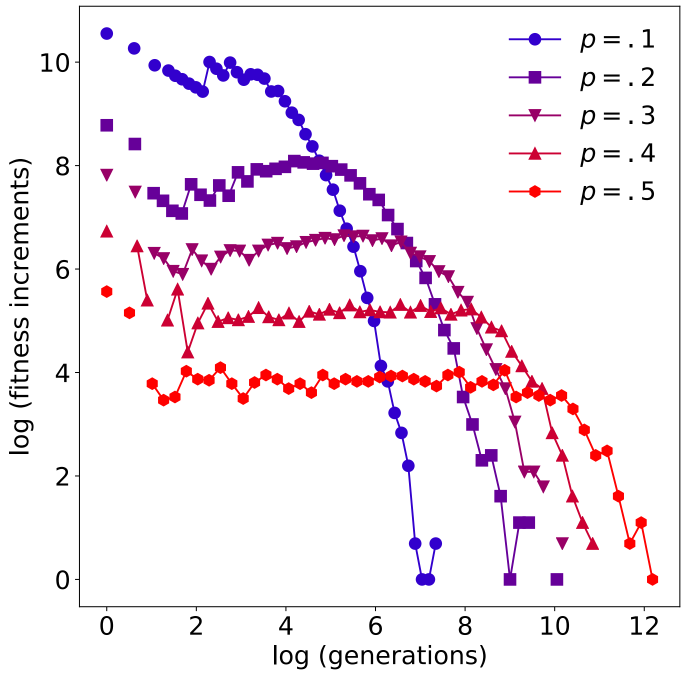

Supplementary Figure S1. Distribution of the periods of stasis (where $\Delta f = 0$) for a range of mutation rates p (simulations as in Fig. 3 and Fig. 4 of the main text). The distribution conforms increasingly to a log-normal distribution for lower mutation rates.

```cpp
/*
 * SUPPLEMENTARY CODE S2
 *
 * C++ PROGRAM FOR GENERATING DATAFILES REQUIRED TO RECREATE FIGURE 4
 * FROM THE MAIN TEXT. SAVE THIS TEXT INTO A FILE WITH A .cpp EXTENSION,
 * e.g., 'evolve.cpp'. COMPILE FROM THE COMMAND LINE USING e.g.,
 * 'g++ -O3 evolve.cpp -o evolve' THEN RUN USING e.g., './evolve' TO
 * GENERATE THE NECESSARY DATA FILES. RUN THE SCRIPT IN S3 TO GENERATE
 * A PLOT.
 *
 * Evolves genome repeatedly according to the fitness function
 * described in the paper associated with this code.
 *
 * Author: S James
 */

#include <iostream>
#include <vector>
#include <set>
#include <sstream>
#include <fstream>
#include <string>
#include <sys/types.h>
#include <unistd.h>
#include <array>

using namespace std;

//! A macro for logging to stdout
#define LOG(s)  cout << "LOG: " << s << endl;

//! Number of genes in a state is set at compile time.
#define N_Genes 5

//! The number of generations to evolve for.
#define N_Generations 100000000

//! 'k=n'
#define N_Ins N_Genes

/*!
 * The genome has a section for each gene. The length of the
 * section of each gene is 2^N_Ins == 2^5 == 32.
 */
typedef unsigned int genosect_t;
#define GENOSECT_ONE 0x1UL

/*!
 * The state has N_Genes bits in it. Working with N_Genes <= 8, so:
 */
typedef unsigned char state_t;

/*!
 * When right-shifting the hi_mask, we need to set the top bit to 1,
 * because right-shifting an unsigned integer number always zero-fills
```

```
 * by default.
 */
#define state_t_top_bit 0x80

/*!
 * Probability of flipping each bit of the genome during evolution.
 */
float pOn;

/*!
 * Starting values of the masks used when computing inputs from a
 * state. When computing input for a gene at position i, the hi_mask
 * is used to obtain inputs for positions > i, the low mask for
 * position < i. The hi bits are then shifted right once to make up an
 * input containing N_Genes-1 bits. Must be set up using masks_init().
 */
//@{
unsigned char lo_mask_start;
unsigned char hi_mask_start;
//@}

/*!
 * The mask used to get the significant bits of genome section. Set up
 * using masks_init().
 */
genosect_t genosect_mask;

/*!
 * The mask used to get the significant bits of a state. Set up using
 * masks_init().
 */
state_t state_mask;

/*!
 * Set the global target states for anterior and posterior positions.
 */
//@{
state_t target_ant = 0x15; // 10101 or 21 dec
state_t target_pos = 0xa;  // 01010 or 10 dec
//@}

/*!
 * Initial anterior and posterior states:
 */
//@{
state_t initial_ant = 0x10; // 10000b;
state_t initial_pos = 0x0;  // 00000b;
//@}

/*!
 * Initialise the masks based on the value of N_Genes
 */
void
masks_init (void)
{
```

```
    // Set up globals. Set N_Ins bits to the high position for the lo_mask
    lo_mask_start = 0x0;
    for (unsigned int i = 0; i < N_Ins; ++i) {
        lo_mask_start |= 0x1 << i;
    }
    hi_mask_start = 0xff & (0xff << N_Genes);

    genosect_mask = 0x0;
    for (unsigned int i = 0; i < (1<<N_Ins); ++i) { // 1<<N is the same as
     2^N
        genosect_mask |= (0x1 << i);
    }

    state_mask = 0x0;
    for (unsigned int i = 0; i < N_Genes; ++i) {
        state_mask |= (0x1 << i);
    }
}

/*!
 * Given a state for N_Genes, and a genome, compute the next
 * state. This is "develop" rather than "evolve".
 */
void
compute_next (const array<genosect_t, N_Genes>& genome, state_t& state)
{
    array<state_t, N_Genes> inputs;

    for (unsigned int i = 0; i < N_Genes; ++i) {
        inputs[i] = ((state << i) & state_mask) | (state >> (N_Genes-i));
    }

    // Now reset state and compute new values:
    state = 0x0;

    // State a anterior is genome[inps[0]] etc
    for (unsigned int i = 0; i < N_Genes; ++i) {
        // Setting state for gene i
        genosect_t gs = genome[i];
        genosect_t inpit = (0x1 << inputs[i]);
        state_t num = ((gs & inpit) ? 0x1 : 0x0);
        if (num) {
            state |= (0x1 << (N_Ins-(i+1)));
        } else {
            state &= ~(0x1 << (N_Ins-(i+1)));
        }
    }
}

/*!
 * Generate a string representation of the state. Something like "1 0 1
 * 1 1" or "0 0 1 1 0".
 */
string
state_str (const state_t& state)
```

```
{
    stringstream ss;
    // Count down from N_Genes, to output bits in order MSB to LSB.
    for (unsigned int i = N_Genes; i > 0; --i) {
        unsigned int j = i-1;
        ss << ((state & (0x1<<j)) >> j) << " ";
    }
    return ss.str();
}

/*!
 * For mixing up bits of three args; used to generate a good random
 * seed using time() getpid() and clock().
 */
unsigned int
mix (unsigned int a, unsigned int b, unsigned int c)
{
    a=a-b;  a=a-c;  a=a^(c >> 13);
    b=b-c;  b=b-a;  b=b^(a << 8);
    c=c-a;  c=c-b;  c=c^(b >> 13);
    a=a-b;  a=a-c;  a=a^(c >> 12);
    b=b-c;  b=b-a;  b=b^(a << 16);
    c=c-a;  c=c-b;  c=c^(b >> 5);
    a=a-b;  a=a-c;  a=a^(c >> 3);
    b=b-c;  b=b-a;  b=b^(a << 10);
    c=c-a;  c=c-b;  c=c^(b >> 15);
    return c;
}

/*!
 * Return a random double precision number between 0 and 1.
 */
double
randDouble (void)
{
    return static_cast<double>(rand()) / static_cast<double>(RAND_MAX);
}

/*!
 * Copy the contents of @from to @to
 */
void
copy_genome (const array<genosect_t, N_Genes>& from, array<genosect_t,
 N_Genes>& to)
{
    for (unsigned int i = 0; i < N_Genes; ++i) {
        to[i] = from[i];
    }
}

/*!
 * The mutation function (which should really be called
 * mutate_genome).
 */
void
```

```
evolve_genome (array<genosect_t, N_Genes>& genome)
{
    for (unsigned int i = 0; i < N_Genes; ++i) {
        genosect_t gsect = genome[i];
        for (unsigned int j = 0; j < (1<<N_Ins); ++j) {
            if (randDouble() < pOn) {
                // Flip bit j
                gsect ^= (GENOSECT_ONE << j);
            }
        }
        genome[i] = gsect;
    }
}

/*!
 * Populate the passed in genome with random bits.
 */
void
random_genome (array<genosect_t, N_Genes>& genome)
{
    for (unsigned int i = 0; i < N_Genes; ++i) {
        genome[i] = ((genosect_t) rand()) & genosect_mask;
    }
}

/*!
 * When working with states in a graph of nodes, it may be necessary
 * to use one bit to refer to the state as being unset; this is the
 * bit to use.
 */
#define state_t_unset 0x80

/*!
 * Evaluates the fitness of one context (anterior or posterior).
 */
double
evaluate_one (array<genosect_t, N_Genes>& genome, state_t state, state_t
 target)
{
    double score = 0.0;

    state_t state_last = state_t_unset;
    set<state_t> visited;
    visited.insert (state); // insert starting state
    for (;;) {
        state_last = state;
        compute_next (genome, state);

        if (visited.count (state)) {
            // Already visited this state so it's a limit cycle
            if (state == state_last) {
                // Point attractor
                if (state == target) {
                    score = 1.0;
                } // else score is definitely 0.
```

```
            } else {
                // Limit cycle

                // Determine the states in the limit cycle by going
                // around it once more.
                set<state_t> lc;
                unsigned int lc_len = 0;
                while (lc.count (state) == 0) {
                    // Check if we have one or both target states on
                    // this limit cycle
                    lc.insert (state);
                    lc_len++;
                    compute_next (genome, state);
                }

                // Now have the set lc; can work out its score.

                // For tabulating the scores
                array<double, N_Genes> sc;
                for (unsigned int j = 0; j < N_Genes; ++j) { sc[j] = 0.0; }

                set<state_t>::const_iterator i = lc.begin();
                while (i != lc.end()) {
                    state_t a = ((*i) ^ ~target) & state_mask;
                    for (unsigned int j = 0; j < N_Genes; ++j) {
                        sc[j] += static_cast<double>( (a >> j) & 0x1 );
                    }
                    ++i;
                }
                // Divide down now.
                for (unsigned int j = 0; j < N_Genes; ++j) {
                    sc[j] /= static_cast<double>(lc_len);
                }

                score = sc[0];
                for (unsigned int j = 1; j < N_Genes; ++j) {
                    score = score * sc[j];
                }
            }
            break;
        }
        visited.insert (state);
    }

    return score;
}

/*!
 * For the passed-in genome, find its final state, starting from the
 * anterior state initial_ant and the posterior state initial_pos
 * (stored in global variables). Return a fitness specifier for the
 * genome.
 *
 * This function examines the limit cycle that is arrived at from the
```

```
 * two initial states. The mean value of each bit in the limit cycle
 * is compared with the target state.
 *
 * The fitness is then computed according
 * to:
 *
 * f = (a0 * a1 * a2 * a3 * a4) * (p0 * p1 * p2 * p3 * p4)
 *
 * a0 is the proportion of time during the limit cycle that bit 0 has
 * the state matching the anterior target
 *
 * Returns fitness in range 0 to 1.0. Note use of double. The fitness
 * values can potentially be very small for a long limit cycle. For
 * example, for a 5 gene LC of size 10, the fitness could be as low as
 * (1/10)^5 * (1/10)^5 = 1/10^10, which is heading towards what a
 * single precision float can represent.
 *
 * For further details on this fitness evaluation, please see the
 * associated paper.
 */
double
evaluate_fitness (array<genosect_t, N_Genes>& genome)
{
    double ant_score = evaluate_one (genome, initial_ant, target_ant);
    double pos_score = evaluate_one (genome, initial_pos, target_pos);
    double fitness = ant_score * pos_score;
    return fitness;
}

/*!
 * A data structure to record information about the fitness increments.
 */
struct geninfo {
    geninfo (unsigned long long int _gen, unsigned long long int _gen_0,
     double _fit)
        : gen(_gen)
        , gen_0(_gen_0)
        , fit(_fit)
        {}
    unsigned long long int gen;   // generations since last increase in
     fitness
    unsigned long long int gen_0; // generation since last F=1
    double fit;                   // The fitness
};

/*
 * For pOn=0.1 to pOn=0.5, perform a loop N_Generations long during
 * which a randomly-selected genome is evolved until a maximally fit
 * (f=1) state is achieved. Once the f=1 state is achieved, the genome
 * is re-randomised and the evolution continues.
 */
int main (int argc, char** argv)
{
    // Initialise masks
    masks_init();
```

```
// Seed the RNG.
unsigned int seed = mix(clock(), time(NULL), getpid());
srand (seed);

for (pOn = 0.1; pOn < 0.6; pOn += 0.1) {

    LOG ("Computing " << N_Generations << " evolutions for p=" << pOn <<
     "...");

    // generations records the relative generation number, and the
    // fitness. Every entry in this records an increase in the fitness
    // of the genome.
    vector<geninfo> generations;

    // Holds the genome and a copy of it.
    array<genosect_t, N_Genes> refg;
    array<genosect_t, N_Genes> newg;

    // The current generation number
    unsigned long long int gen = 0;
    // The last generation to have seen a fitness increment
    unsigned long long int lastgen = 0;
    // The last generation at which there was an f=1 genome
    unsigned long long int lastf1 = 0;
    // A count of f=1 genomes to print out at the end
    unsigned long long int f1count = 0;

    while (gen < N_Generations) {

        // At the start of the loop, and every time fitness of 1.0 is
        // achieved, generate a random genome starting point.
        random_genome (refg);
        ++gen; // Because we randomly generated.

        // Evaluate the fitness of the initial, reference genome.
        double a = evaluate_fitness (refg);

        // a randomly selected genome can be maximally fit
        if (a==1.0) {
            generations.push_back (geninfo(gen-lastgen, gen-lastf1, a));
            lastgen = gen;
            lastf1 = gen;
            ++f1count;
        }

        // Test fitness to determine whether we should mutate or
        // loop back and re-randomise to start a new cycle.
        while (a < 1.0) {

            // Copy the genome
            copy_genome (refg, newg);
            // Mutate the copy
            evolve_genome (newg);
            ++gen; // Because we mutated
```

```
        if (gen > 0 && (gen % 1000000 == 0)) {
            LOG ("[pOn=" << pOn << "] That's " << gen/1000000.0 <<
             "M generations (out of "
                 << N_Generations/1000000.0 << "M) done...");
        }

        // Is it time to break out of the loop?
        if (gen >= N_Generations) {
            break;
        }

        // Evaluate the fitness of the mutated copy
        double b = evaluate_fitness (newg);

        if (b < a) {
            // Mutated genome is less fit; do nothing else
        } else {
            // Mutated genome is as fit or fitter, so record
            // the fitness increase in generations
            generations.push_back (geninfo(gen-lastgen, gen-lastf1,
             b));
            lastgen = gen;
            if (b==1.0) {
                lastf1 = gen;
                ++f1count;
            }
            // update the current, best fitness stored in 'a'
            a = b;
            // and copy the new, fitter, mutated genome to refg
            copy_genome (newg, refg);
        }
    }
}

LOG ("p=" << pOn << ". Generations size: " << generations.size() <<
 " with " << f1count << " F=1 genomes found.");

// Save data from memory (vector<geninfo> generations) out to a
 file.
ofstream f;
stringstream pathss;
pathss << "./";
pathss << "evolve_";
pathss << "a" << (unsigned int)target_ant << "_p" << (unsigned
 int)target_pos;
pathss << "_ff4_" << N_Generations << "_gens_" << pOn << ".csv";

f.open (pathss.str().c_str(), ios::out|ios::trunc);
if (!f.is_open()) {
    cerr << "Error opening " << pathss.str() << endl;
    return 1;
}

for (unsigned int i = 0; i < generations.size(); ++i) {
```

```
            // In the file is recorded the time taken to get to F=1
            if (generations[i].fit == 1.0) {
                f << generations[i].gen_0 << endl;
            }
        }
        f.close();
    }

    return 0;
}
```

```python
'''
SUPPLEMENTARY CODE S3

PYTHON SCRIPT FOR RECREATING FIGURE 4 FROM THE MAIN TEXT
SAVE THIS TEXT INTO A FILE WITH A .PY EXTENSION, E.G. 'plot.py'.
FIRST RUN THE S2 PROGRAM TO GENERATE THE NECESSARY DATAFILES.
THEN RUN FROM THE SAME DIRECTORY USING E.G. 'python plot.py'
'''

import numpy as np
import matplotlib
matplotlib.use ('TKAgg', warn=False, force=True)
import matplotlib.pyplot as plt
import sys
import csv

driftnodrift = 'drift'
ff='ff4'

# Read csv files.
def readDataset (filepath):
    f = np.zeros([1,1])
    with open (filepath, 'r') as csvfile:
            rdr = csv.reader (csvfile)
            for row in rdr:
                f[-1] = float(row[0])
                f = np.append(f, np.zeros([1,1]), 0)
    # Note the -1 as there will be a final, zero line in the array
    return f[:-1,:]

maxgens='100000000'
filetag = ''
files = ['./evolve_a21_p10_'+ff+'_'+maxgens+'_gens_0.1.csv',
         './evolve_a21_p10_'+ff+'_'+maxgens+'_gens_0.2.csv',
         './evolve_a21_p10_'+ff+'_'+maxgens+'_gens_0.3.csv',
         './evolve_a21_p10_'+ff+'_'+maxgens+'_gens_0.4.csv',
         './evolve_a21_p10_'+ff+'_'+maxgens+'_gens_0.5.csv']
nf = len(files)

lbls = ['p=0.10', 'p=0.20', 'p=0.30', 'p=0.40', 'p=0.50']
mkr = ['o', 'v', 's', '^','h']
ms = [8, 9, 8, 9, 8]

# Font size for plotting
fs=20

# Set a default fontsize for matplotlib:
fnt = {'family' : 'DejaVu Sans',
       'weight' : 'regular',
       'size'   : fs}
matplotlib.rc('font', **fnt)

scale = 1000.

f1 = plt.figure(figsize=(17,8)) # Figure object
```

```
a1 = f1.add_subplot (1,2,1)
a2 = f1.add_subplot (1,2,2)

M = np.zeros([nf,3])

# Global choice of nbins
nbins_g = 25

# Holds lines so that legends can be made
lines = []
fcount = 0

# Plot one point only for each set; this is what will give us the
# legend looking sensible, with both a point and a line. This is a
# dirty hack! Complain to matplotlib legend developers.
for y,fil in enumerate(files):
    fcount = fcount+1
    nbins = nbins_g
    D = readDataset (fil)
    if D.size == 0:
        continue
    bins = np.linspace(1,0.5*np.max(D),nbins)
    h,b = np.histogram (D, bins)
    # Plot points
    colo = plt.cm.brg((fcount*0.5)/len(files))
    pp = a1.plot(b[0]/scale,np.log(h)
     [0],'.-',color=colo,marker=mkr[y],markersize=ms[y])

fcount = 0
# Plot points proper
for y,fil in enumerate(files):
    print ('Processing file: {0}'.format(fil))
    fcount = fcount+1
    nbins = nbins_g
    D = readDataset (fil)
    if D.size == 0:
        continue

    # linlog
    bins = np.linspace(1,0.5*np.max(D),nbins)
    h,b = np.histogram (D, bins)
    colo = plt.cm.brg((fcount*0.5)/len(files))
    pp = a1.plot(b[:-1]/
     scale,np.log(h),'.',color=colo,marker=mkr[y],markersize=ms[y])

    # loglog
    bins = np.logspace (np.log10(np.min(D)), np.log10(np.max(D)), base=10,
     num=nbins)
    h,b = np.histogram (D, bins)
    colo = plt.cm.brg((fcount*0.5)/len(files))

     a2.plot(np.log10(b[:-1]),np.log10(h),'.-',color=colo,marker=mkr[y],mark
     ersize=ms[y])
```

```python
# Plot the best fit lines.
fcount = 0
printlines = 1
for y,fil in enumerate(files):
    print ('Processing file: {0}'.format(fil))
    fcount = fcount+1
    nbins = nbins_g
    D = readDataset (fil)
    print ('D has rank {0}, shape {1} and size {2}'.format(D.ndim, D.shape,
     D.size))
    if D.size == 0:
        continue
    bins = np.linspace(1,0.5*np.max(D),nbins)
    h,b = np.histogram (D, bins)

    # Do a fit
    x = np.where(np.log(h)>0)[0]
    print ('x: {0}'.format(x))
    print ('x size: {0}'.format(x.size))
    if x.size > 0:
        bx = b[x]/scale
        print ('b: {0}'.format(b))
        print ('h: {0}'.format(h))
        fit = np.polyfit (bx, np.log(h[x]), 1)
        fit_fn = np.poly1d (fit)

        # Slope is fit[0], Record for a later graph.
        M[y,0] = fit[0]     # slope
        colo = plt.cm.brg((fcount*0.5)/len(files))
        if printlines:
            print ('Plotting file {0}'.format(fil))
            ll = a1.plot(bx,fit_fn(bx),'-',linewidth=2,color=colo)

    else:
        M[y,0] = 0     # no slope known

    M[y,1] = (y+1)*0.05 # p, the flip probability.
    M[y,2] = np.mean(D) # mean generations, in 10K

a1.legend(lbls,frameon=False)
a1.set_ylabel(r'log (evolutions)',fontsize=fs)
a1.set_xlabel('1000 generations',fontsize=fs)
a1.set_ylim([0,10])
a1.set_xlim([-5,100])
a1.set_axisbelow(True)

a2.legend(lbls,frameon=False)
a2.set_ylabel(r'log$_{10}$ (evolutions)',fontsize=fs)
a2.set_xlabel('log$_{10}$ (generations)',fontsize=fs)
#a2.set_ylim([0,10])
#a2.set_xlim([-5,100])
a2.set_axisbelow(True)

# Slope vs p fit. Fit line to most of the points.
slope_fit = np.polyfit (M[1:,1], M[1:,0]/scale, 1)
```

```
slope_fit_fn = np.poly1d (slope_fit)

f1.tight_layout()
plt.savefig ('figure.svg')

plt.show()
```
